# Factors Predicting Ambulatory Status at Discharge After Fragility Hip Fracture Surgery: A Retrospective Cohort Study

**DOI:** 10.3390/medsci14010017

**Published:** 2025-12-30

**Authors:** Thitirut Jongutchariya, Palanthorn Loomcharoen, Jittima Saengsuwan, Saowaluck Settheekul

**Affiliations:** 1 Department of Rehabilitation, Hatyai Hospital, Songkhla 90110, Thailand; jthitirutpmr@cpird.in.th (T.J.); ploomcharoen.pmr@cpird.in.th (P.L.); 2 Department of Rehabilitation Medicine, Faculty of Medicine, Khon Kaen University, Khon Kaen 40002, Thailand; 3 Independent Researcher, Chiang Mai 50300, Thailand; rai.saowaluck@gmail.com

**Keywords:** ambulatory recovery, functional ability, hip arthroplasty, older adults, rehabilitation, walking

## Abstract

**Background**/**Objectives**: Ambulatory status at hospital discharge contributes to subsequent functional recovery in older adults following hip fracture. This study aimed to identify independent predictors of ambulatory status at hospital discharge following surgery for fragility hip fractures in a tertiary care setting in Southern Thailand. **Methods**: A retrospective study was conducted among patients aged 50 years and older who underwent surgery for low-energy hip fractures between 1 October 2018, and 30 September 2023. Data on preoperative, intraoperative, postoperative, and process of care factors were collected from electronic medical records. Student’s *t*-tests and chi-square tests compared candidate variables between groups. Univariable and multivariable risk analyses were performed to identify independent predictors of ambulation at discharge. **Results**: Among 532 patients (72.7% women; mean age 76.8 ± 9.7 years), 314 (59.0%) were ambulatory at hospital discharge. Multivariable analysis demonstrated that achieving rehabilitation at the ambulation training level (mRR = 24.10; 95% CI: 9.14–63.60; *p* < 0.001) and undergoing hip arthroplasty (mRR = 1.17; 95% CI: 1.07–1.29; *p* < 0.001) were significant positive predictors of ambulation. Conversely, a history of cerebrovascular disease with hemiplegic sequelae (mRR = 0.70; 95% CI: 0.53–0.91; *p* < 0.01) and delayed initiation of rehabilitation more than 72 h postoperatively ((mRR = 0.84; 95% CI: 0.73–0.97; *p* < 0.05) were associated with reduced likelihood of ambulation. **Conclusions**: Ambulatory status at hospital discharge was strongly associated with early, ambulation-level rehabilitation and hip arthroplasty, whereas history of stroke and delayed rehabilitation reduced mobility. These findings emphasize the importance of timely, targeted rehabilitation to optimize functional recovery after hip fracture surgery.

## 1. Introduction

Global demographic shifts, marked by population aging and declining fertility necessitate prompt societal adaptation [1]. Consequently, aging is associated with an increased risk of multiple comorbidities. Among these, musculoskeletal disorders such as osteoporotic fragility hip fractures have emerged as a significant public health concern, substantially contributing to elevated rates of morbidity and mortality, functional decline, reduced quality of life (QOL), and escalating healthcare costs among older adults [2,3]. In 2019, the global incidence of hip fractures reached 14.2 million, a 92.7% increase since 1990, disproportionately affecting females and with nearly half occurring in individuals aged 85 years and older. Projections indicate an approximate 1.9-fold rise by 2050, with a more pronounced increase among males (2.4-fold) compared to females (1.7-fold) [4]. In Asia, the number of cases is expected to rise from 1.1 million in 2018 to 2.6 million by 2050, largely driven by demographic changes in China and India [5], whereas in Thailand, the age-standardized incidence rate showed a slight decline to 140.7 per 100,000 in 2022 [6]. Despite advances in surgical technique, implant technology, and multidisciplinary care, hip fracture in older adults remains the most serious type of fracture, with 1-year mortality rates ranging from 15% to 36% [7].

Currently, surgical management remains the standard treatment for clinically stable patients with fragility hip fractures, which are generally defined as fractures occurring in adults aged ≥ 50 years resulting from low-energy mechanisms [8,9]. The primary objective following hip surgery is to restore patients to their pre-fracture status, with an emphasis on regaining optimal ambulatory function and basic self-care abilities. Achieving these complex outcomes requires a comprehensive and multidisciplinary approach to care. Evidence from previous studies has demonstrated that the recovery of ambulation and favorable functional outcomes are influenced by multiple interrelated factors. These include sociodemographic variables (e.g., age, sex, body mass index (BMI), smoking) [10,11,12,13,14,15,16], baseline pre-fracture ambulatory status (e.g., independent community ambulation) [10,11,12,16], and existing comorbidities (e.g., cognitive impairment, hemiplegia, chronic kidney disease (CKD), osteoporosis) [10,11,12,15,16,17,18,19], and higher American Society of Anesthesiologists (ASA) scores [10,11]. Additionally, fracture- and surgery-related factors including delayed surgical intervention (e.g., beyond 48 h) and the specific type of hip procedure, along with admission laboratory parameters (e.g., serum albumin and hematocrit levels), significantly influence postoperative outcomes [10,11,16,19]. Postoperative complications, including intensive care unit (ICU) admission, weight-bearing restrictions, prolonged urinary catheterization, pressure ulcers, pneumonia, and urinary tract infections (UTIs), can further impede recovery [10,11,16,20,21]. Finally, system-level factors, including the comprehensiveness of the rehabilitation program, particularly the timing of initiation and the targeting of appropriate program training, as well as the total length of hospital stay, are important determinants of both short-term and long-term ambulatory and functional outcomes in patients.

However, a critical gap remains in the existing literature. Most studies have analyzed prognostic factors in isolation or focused predominantly on preoperative variables and long-term mortality. There is a paucity of research integrating the entire surgical continuum—encompassing preoperative, intraoperative, postoperative, and process of care factors—into a single predictive model for short-term functional recovery. Specifically, the impact of the precise timing of rehabilitation initiation on discharge ambulatory status remains understudied. To address this limitation, this study aimed to identify predictors of ambulatory status at hospital discharge among older adults undergoing surgery for fragility hip fractures. By examining factors across all phases of care, this study seeks to inform clinical decision-making and optimize care strategies to enhance functional outcomes for future patients.

## 2. Materials and Methods

### 2.1. Design and Setting

This prognostic prediction research was conducted using a retrospective observational cohort design at a tertiary care referral center, Hatyai Hospital, Songkhla, Thailand.

Data were obtained from electronic medical records (EMRs) between 1 October 2018, and 30 September 2023. From an initial pool of 821 patients identified using the International Classification of Diseases, 10th Revision (ICD-10) codes for hip fracture, those meeting the eligibility criteria were included. The inclusion criteria were (1) age ≥ 50 years; (2) diagnosis of a single, closed hip fracture resulting from low-energy trauma; (3) fracture categorized as femoral neck, intertrochanteric, or subtrochanteric, according to the corresponding ICD-10 codes (S72.000–S72.019, S72.100–S72.101, S72.110–S72.111, and S72.200–S72.210); and (4) receipt of surgical treatment for hip fracture, identified using ICD-9 procedure codes (79.15, 79.25, 79.35, 81.51, and 81.52). The exclusion criteria were (1) conservative (non-surgical) management; (2) hip fractures secondary to pathological conditions (e.g., malignancy); (3) multiple traumatic injuries sustained during the same admission; (4) fractures resulting from high-energy trauma; (5) periprosthetic fractures or fractures involving prior nail fixation; (6) pre-fracture bedridden status or wheelchair dependence; (7) in-hospital mortality before discharge; and (8) incomplete or missing EMR data during the study period.

Surgical interventions were performed according to fracture type. Postoperatively, patients adhered to a standardized functional training protocol established by a Physical Medicine and Rehabilitation (PM&R) Physician. This program aimed to restore ambulatory function, with the protocol being tailored to accommodate individual physical status, comorbidities, surgical procedure, and pain tolerance. Rehabilitation emphasized early mobilization, consisting of daily 20- to 30-min sessions delivered in the inpatient ward. These sessions integrated three core components: mobility exercise, transfer training, and ambulatory training. Specific activities included bed mobility exercises, supine-to-sitting transitions, sitting balance training, sit-to-stand maneuvers, pivot transfers, and standing balance training. Ambulation training with walking practice was initiated once patients demonstrated stability, and they were encouraged to use a walker and practice partial weight bearing on the surgical limb as tolerated.

### 2.2. Ethical Considerations

The study was approved by the Human Research Ethics Committee of Hatyai Hospital (Approval No. HYH EC 007-67-01, date of approval 6 February 2024). Informed consent was waived by the Ethics Committee in accordance with regulatory and ethical guidelines for retrospective studies.

### 2.3. Sample Size Calculation

For this observational study, the minimum required sample size was determined according to the recommendation of Bujang et al. [22], indicating that a sample size of 500 participants is adequate to ensure reliable parameter estimation and reduce bias in regression analysis.

### 2.4. Study Endpoint

The primary endpoint of this study was ambulatory status at hospital discharge following surgery for fragility hip fractures. Patients were categorized into two groups based on their discharge ambulation status: (1) an ambulatory group, defined as patients who were able to walk independently with a gait aid, and (2) a non-ambulatory group, defined as patients who were either bedridden or dependent on a wheelchair for mobility.

### 2.5. Data Collection

Data extraction was conducted by the first and second authors and cross-verified to ensure accuracy and consistency. Data on candidate predictors were collected using a case record form developed through an extensive literature review and categorized into four domains. This stratification enabled the identification of phases of care that were most likely to be effective.

**Preoperative factors** included demographic and clinical characteristics, as well as active medical problems at admission. Variables comprised the followings: age [13,14,15]; sex [10,11,12,16]; BMI [10,11,12,16]; smoking status [11]; comorbidities [10,11,12,15,16,17,18,19] such as history of stroke with residual hemiplegia [17], dementia [18], osteoporosis [10,11], sarcopenia [10], and CKD [19]; total number of medications used [23]; baseline pre-fracture ambulatory status [10,11,12,16,19,24,25]; fracture-related characteristics [10,11,16,26] (cause and location of injury, fracture type, number of fractures); pertinent laboratory parameters [10,11,16,19,25]; and other active medical conditions present at admission [27,28].

**Intraoperative factors** encompassed surgical and perioperative variables, including time from hospital admission to surgery [10,11,29]; type of operation [10,11]; ASA physical status classification [10,11]; duration of surgery and anesthesia [10,11,16]; type of anesthesia administered [11,14,20]; estimated intraoperative blood loss [16]; and intraoperative surgical or anesthetic complications [16,20,30,31].

**Postoperative factors** comprised clinical events, medical management, and rehabilitation-related parameters. These included ICU admission or ventilator use [16,32] (defined as either ICU admission irrespective of ventilator use or endotracheal intubation with mechanical ventilation in any hospital setting); postoperative need for oxygen supplementation [20]; urinary catheterization on postoperative day 2 [16,33]; surgical restrictions on ambulation [11,20]; pain scores on the rehabilitation day [16]; anemia attributable to acute postoperative blood loss [34]; occurrence of other postoperative medical complications [20,21]; and time from surgery to initiation of functional training [35,36,37]. Functional training was defined as structured rehabilitation activities, comprising mobility exercises, transfer, and ambulation training [38,39]. Additionally, process-of-care measures included time to internal medicine consultation [40] and total length of hospital stay (LOS) [10,25,41].

### 2.6. Data Analysis

Statistical analysis was conducted using Stata Statistical Package version 18.0 (Stata Corp LLC, College Station, TX, USA). Categorical variables were summarized as frequencies and percentages, while continuous variables were reported as means and standard deviations (SD) for normally distributed data or medians and interquartile ranges (IQR) for skewed data. Group comparisons between ambulatory and non-ambulatory patients were conducted using the Chi-square test for categorical variables; Fisher’s exact test was applied when expected cell counts were less than five. Continuous variables were compared using Student’s *t*-test or the Mann–Whitney U test, depending on the distribution of the data. Any variables with more than 50% missing data were not considered potential predictive factors and were not included in the analysis.

Factors with a *p*-value < 0.25 and those deemed clinically relevant were included in a univariable regression analysis [42]. The collinearity of each candidate predictor was assessed. Predictors which had a variant inflation factor (VIF) value > 5 were excluded from the multivariable analysis [43]. Multivariable regression analysis using a Poisson distribution with robust standard errors [44] was conducted to comprehensively assess associations between predictive factors and ambulation status at hospital discharge following fragility hip fracture surgery. Results were reported as univariable risk ratios (uRR) and multivariable risk ratio (mRR) with corresponding 95% confidence intervals (CI) and *p*-values. A two-sided *p*-value < 0.05 was considered statistically significant.

## 3. Results

### 3.1. Study Population and Exclusions

**Figure 1:**
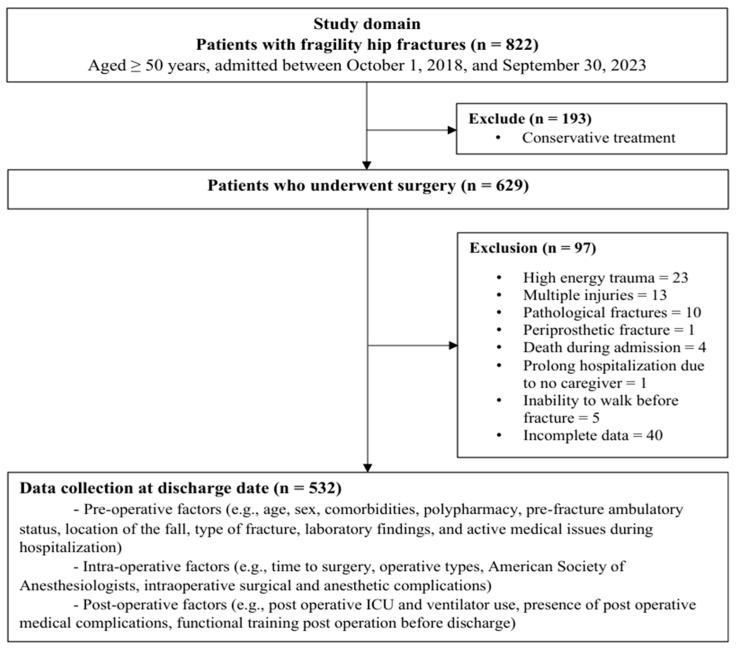
Flow diagram of patients in this study.

Of the 822 patients initially screened, 629 patients had sustained fragility hip fractures and underwent surgical intervention, while 193 were managed conservatively. A total of 97 patients were excluded due to high-energy trauma, multiple injuries, pathologic fractures, periprosthetic fractures, pre-operative in-hospital mortality, prolonged hospitalization, inability to ambulate prior to the fracture, and incomplete data. As a result, 532 patients met the inclusion criteria and were included in the final analysis (Figure 1). At the time of hospital discharge, 314 patients (59.0%) had regained ambulatory ability, whereas 218 (41.0%) remained non-ambulatory.

### 3.2. Patient Characteristics

The final cohort consisted of 532 patients (age: 76.8 ± 9.7 years; 72.7% female). The most prevalent comorbidities were hypertension (69.2%), CKD (34.6%), and cerebrovascular disease (18.0%). Polypharmacy, defined as the concurrent use of five or more medications, was observed in approximately one-quarter of the population. Regarding pre-fracture ambulatory status, more than half of the patients were independent community ambulators without gait aids (61.3%). The primary mechanism of injury was a low-energy fall occurring within an indoor environment. Closed femoral neck fractures were the predominant fracture type (49.4%), with surgical fixation being the most common intervention (56.8%). In terms of care processes, 38.5% of patients underwent surgical fixation within 72 h of admission. The median LOS was 10 days, and the median Barthel Index score [45] at discharge was 9 out of 20, indicating moderate to severe functional dependence consistent with early postoperative limitations in mobility and self-care (Table 1).

### 3.3. Comparison of Predictors Between Ambulatory and Non-Ambulatory Patients

As detailed in Table 2, univariate analysis revealed distinct differences in preoperative, intraoperative, and postoperative characteristics between the two groups at hospital discharge. Regarding preoperative factors, patients in the non-ambulatory group were significantly older than those in the ambulatory group (*p* < 0.001), whereas gender distribution, BMI, and smoking status were comparable. The non-ambulatory group exhibited a significantly higher burden of comorbidities, notably CKD (*p* < 0.05), and heart disease (*p* < 0.01), along with a higher rate of antiplatelet medication usage (*p* < 0.01). Conversely, pre-fracture independent community ambulation (without gait aids) and admission laboratory parameters, specifically hematocrit (Hct) and estimated glomerular filtration rate (eGFR), were significantly higher in the ambulatory group (*p* < 0.001, *p* < 0.001, and *p* < 0.01, respectively). Furthermore, the non-ambulatory group experienced a significantly higher incidence of in-hospital complications, including acute kidney injury (AKI), new-onset anemia, hypokalemia, hyperkalemia, UTIs, delirium, pneumonia, and COVID-19 infection (*p* < 0.05 for all).

In terms of intraoperative factors, early surgery (<72 h), hip arthroplasty, and the use of regional anesthesia were significantly more frequent in the ambulatory group (*p* < 0.001, *p* < 0.001, and *p* < 0.05, respectively). In contrast, intraoperative anemia and an ASA classification of III–IV were significantly more prevalent in the non-ambulatory group (*p* < 0.05 and *p* < 0.001, respectively).

Regarding postoperative factors, the non-ambulatory group demonstrated a greater requirement for ICU admission, oxygen support, and urinary catheterization, as well as a higher incidence of pressure ulcers, delirium, and UTIs (*p* < 0.01 for all). Crucially, rehabilitation timing differed significantly: the non-ambulatory group was associated with a delayed initiation of functional training (*p* < 0.001) and lower participation rates in ambulation training sessions compared to the ambulatory group (*p* < 0.001).

### 3.4. Predictive Factors for Ambulation

The results of the univariable and multivariable regression analyses are presented in Table 3. While univariable analysis revealed multiple factors associated with ambulatory status at hospital discharge, the multivariable model identified four key predictors. Participation in ambulation training (mRR = 24.10; 95% CI: 9.14–63.60; *p* < 0.001) and undergoing hip arthroplasty (mRR = 1.17; 95% CI: 1.07–1.29; *p* < 0.001) emerged as significant positive predictors of ambulatory capacity at discharge. Conversely, a history of stroke (mRR = 0.70; 95% CI: 0.53–0.91; *p* < 0.01) and delayed rehabilitation initiation (>72 h postoperatively; mRR = 0.84; 95% CI: 0.73–0.97; *p* < 0.05) were significant negative predictors.

## 4. Discussion

Older adults with hip fractures frequently experience impaired basic activities of daily living (ADLs), reduced mobility, increased morbidity and mortality, and diminished QOL [46]. Consequently, restoring pre-fracture ambulatory status remains a primary goal of hip fracture management, serving as a pivotal benchmark for functional recovery and a critical outcome for both patients and caregivers [47]. Early restoration of ambulation is notably associated with significantly higher survival rates at both one and ten years postoperatively [48]. In our cohort, 59.0% of patients regained ambulatory capacity at discharge, a rate falling within the broad range reported globally (20% to 79.5%) [16,25,49,50], reflecting satisfactory recovery of ambulatory function. Adulkasem et al. [25] highlighted the prognostic value of discharge status, demonstrating that inability to walk at discharge significantly predicts mobility impairment at three months postoperatively (multivariable odds ratio [mOR] = 3.39; 95% CI: 1.6–7.5). These findings underscore the importance of early multidisciplinary rehabilitation aimed at achieving ambulation to optimize functional outcomes in older adults with hip fractures.

In this study, we developed a multivariable model integrating preoperative, intraoperative, and postoperative factors to identify independent predictors of ambulatory status. The analysis revealed that hip arthroplasty, a history of stroke, delayed rehabilitation initiation, and participation in ambulation training were significant predictors of ambulatory capacity at discharge. Regarding surgical intervention, undergoing hip arthroplasty significantly increased the likelihood of discharge ambulation compared to fixation. This finding supports both local and international literature, reporting that undergoing hip arthroplasty was a significant positive predictor of ambulatory status [11,24,51,52] and lessens the length of generic rehabilitation stay [53] in this group of patients. In line with Villaumbrosia et al. [11], hip arthroplasty, particularly via the posterior approach with cemented hemiarthroplasty, facilitates early weight bearing and timely ambulation training. This approach enhances walking ability, restores pre-fracture ambulation, shortens hospital stays, and reduces complications such as pneumonia, pressure ulcers, and deep vein thrombosis [54,55]. Conversely, partial or delayed weight bearing strategies are associated with slower recovery, prolonged immobility, increased medical complications, and length of hospital stays, highlighting the need for individualized ambulation training based on fracture type, surgical approach, and bone quality [56].

Neurological comorbidities exert a profound influence on functional trajectories. A history of stroke with residual hemiplegia is commonly associated with gait disturbances and reduced pre-fracture ambulatory capacity, with age-related physiological decline further exacerbating these mobility limitations. Our results align with previous Thai studies indicating that patients with pre-existing neurological impairments or cognitive deficits face substantial challenges in achieving independent walking [16,19]. Similarly, international studies demonstrated that a history of stroke substantially impairs postoperative ambulatory recovery [10,11,12,15,17]. These findings necessitate risk-stratified care pathways. Clinicians must recognize the limited recovery potential in this subgroup and implement targeted rehabilitation interventions aimed at facilitating early postoperative mobilization and ensuring a safe transition to the home environment.

Regarding rehabilitation timing, initiation of functional training, particularly weight bearing within 24 to 48 h postoperatively, is strongly recommended in geriatric patients. It represents a cornerstone of postoperative rehabilitation, supported by guidelines from the American Academy of Orthopedic Surgeons (AAOS) [57], the National Institute for Health and Care Excellence (NICE) [58], and clinical experience in Thailand [39], owing to its association with improved physical and mental function and reduced risk of thromboembolism and pulmonary complications [39,59,60]. Conversely, delayed mobilization is linked to higher rates of medical complications, prolonged hospital stays, and increased mortality. Accordingly, the timing of functional mobility training has emerged as a crucial factor influencing postoperative recovery. In this study, initiation of rehabilitation more than 72 h postoperatively was a significant negative predictor of ambulatory status at hospital discharge. This finding contrasts with Nakamura et al. [38], which reported that early ambulation on postoperative day 1 or 2 was associated with independent walking at 1 week (OR = 3.27; 95% CI 2.17–4.94; *p* < 0.0001) and at hospital discharge (OR = 3.33; 95% CI 2.38–4.69; *p* < 0.0001). Together, these findings support previous studies demonstrating that early mobilization improves functional outcomes and enhances 30-day survival after hip fracture surgery [36,37,61]. Our findings further emphasize the importance of prompt intervention by defining a critical therapeutic window during which initiating mobility training may optimize recovery. Given the trend toward reduced hospital length of stay, implementing structured early mobilization protocols is increasingly essential to ensure optimal postoperative outcomes and efficient use of rehabilitation resources.

Notably, the strongest predictor in our model was the successful participation in ambulation training. This variable likely serves a dual function: as a therapeutic intervention and as a proxy for physiological reserve, implying that patients capable of engaging in intensive training possess greater baseline vitality. Identifying this milestone allows clinicians to distinguish between failures due to system-level delays (initiation) versus those limited by patient-level physiological capacity (training completion). Comparing different rehabilitation modalities, a recent scoping review identified that strength-training programs and multidisciplinary rehabilitation (delivered in nursing homes) provide the most robust evidence for enhancing ambulatory status within one month to one year post hip fracture surgery [11]. Furthermore, optimizing the intensity of therapy within these multidisciplinary pathways is suggested to facilitate faster functional recovery [53]. While our findings underscore the value of in-hospital mobility, transfer, and ambulation training, integrating these intensive modalities into a post-discharge care plan could further sustain and enhance the recovery trajectory initiated during hospitalization.

### 4.1. Strengths

This study has several notable strengths. It comprehensively evaluates both modifiable and nonmodifiable factors influencing ambulatory status at hospital discharge after fragility hip fracture surgery in older adults. By integrating preoperative, intraoperative, postoperative, and system-level care processes, including comorbidities, fracture type, surgical procedure, weight bearing status, and the timing of initiation of functional and ambulation-level rehabilitation training, the study provides a multidimensional perspective on functional recovery. The inclusion of detailed postoperative rehabilitation parameters highlights a critical therapeutic window for early mobilization. Collectively, these insights enhance the clinical relevance of the study and clarify how coordinated care across the surgical continuum influences early mobility recovery, particularly ambulatory status at hospital discharge, with important implications for clinical decision-making and geriatric care in patients undergoing fragility hip fracture surgery.

### 4.2. Limitations

This study has several limitations. First, its retrospective design and reliance on EMRs may have introduced missing or inaccurate data, despite careful review. Second, as the study was conducted at a single hospital in Southern Thailand, the findings may have limited generalizability to other populations or settings with different demographics, surgical practices, or rehabilitation protocols. Third, although multivariable analysis adjusted for known confounders, the potential for residual confounding remains, particularly regarding unmeasured variables such as nutritional status, social support, and patient motivation. Additionally, specific geriatric syndromes such as sarcopenia and frailty, as well as the severity of osteoporosis, were not consistently documented in the routine EMRs, precluding their inclusion as specific variables in the analysis. Fourth, the association between achieving rehabilitation at the ambulation training level and ambulatory status at hospital discharge exhibited a wide 95% confidence interval (mRR 24.10; 95% CI: 9.14–63.60), reflecting uncertainty in the effect size likely due to a limited number of patients reaching that rehabilitation level or data variability, warranting cautious interpretation. Finally, the outcome measure was limited to ambulatory status at hospital discharge, representing an early stage of recovery, and did not assess long-term functional outcomes. Future research should include larger, more diverse populations across multi-site cohorts and employ validated functional mobility assessments over extended follow-up periods to better understand the factors influencing recovery after hip fracture surgery.

## 5. Conclusions

This study successfully identified key preoperative, intraoperative, and postoperative factors predicting ambulatory status at discharge following fragility hip fracture surgery. Our findings demonstrate that achieving rehabilitation at the ambulation training level and undergoing hip arthroplasty are significant independent positive predictors. Conversely, a history of stroke and delayed initiation of rehabilitation (>72 h postoperatively) are associated with poorer ambulatory outcomes. These results emphasize the critical role of early and targeted rehabilitation interventions, as well as surgical choice, in optimizing functional recovery. Incorporating these factors into clinical practice may enhance risk stratification and inform the development of tailored treatment strategies aimed at improving postoperative mobility and patient outcomes.

## Figures and Tables

**Table 1:** Patient demographics and clinical characteristics (n = 532).

Variables	Number	Percent
Age (years, mean ± SD)	76.8 ± 9.7	
Female	387	72.7
Comorbidities		
-Hypertension	368	69.2
-Chronic kidney disease	184	34.6
-Cerebrovascular disease	96	18.0
-Musculoskeletal problems	67	12.6
-Anemia	63	11.8
-Heart disease (AF/VHD/IHD)	51	9.6
-Pulmonary disease	60	11.3
-Alzheimer/Dementia	29	5.5
Polypharmacy (Use of ≥5 medications)	154	28.9
Indoor (falling area)	434	81.6
Fall from slipping (cause of fracture)	459	86.3
Ambulatory status before fracture		
-Home walk, with gait aids	105	19.7
-Home walk, no gait aids	60	11.3
-Community walk, with gait aids	41	7.7
-Community walk, no gait aids	326	61.3
Close fracture neck of femur	263	49.4
Type of operation		
-Fixation (multiple screw/plate and screw/intramedullary nail)	302	56.8
-Arthroplasty	230	43.2
Time from admission to internal medicine consultation (days, median (IQR))	1 (1)	
Surgery within 72 h from admission	205	38.5
Length of stay (days, median (IQR))	10 (4)	
Barthel Index at discharge (scores, median (IQR))	9 (3)	

Mann-Whitney U test. Abbreviations AF, atrial fibrillation; IHD, ischemic heart disease; VHD, valvular heart disease; IQR, interquartile range; SD, standard deviation.

**Table 2:** Comparison of preoperative, intraoperative, and postoperative clinical characteristics between ambulatory and non-ambulatory patients at discharge following fragility hip fracture surgery (n = 532).

Characteristics	Ambulatory	Non-Ambulatory	*p*-Value
	(n = 314)	(n = 218)	
**Preoperative factors**			
Female	230 (73.2)	157 (72.0)	0.754
Age ≥ 80 years	111 (35.4)	203 (93.1)	<0.001
Body mass index (kg/m^2^, mean ± SD)	22.5 ± 4.1	22.3 ± 3.9	0.480
Smoking	31 (9.9)	22 (10.1)	0.934
Comorbidities			
-Hypertension	210 (66.9)	158 (72.5)	0.169
-Chronic kidney disease	98 (31.2)	86 (39.4)	0.049
-Musculoskeletal problems (Osteoporosis/Lumbar spondylosis)	35 (11.1)	32 (14.7)	0.227
-Cerebrovascular disease	17 (5.4)	21 (9.6)	0.063
-Anemia	32 (10.2)	31 (14.2)	0.157
-Pulmonary disease	30 (9.6)	30 (13.8)	0.131
-Heart diseases (IHD/VHD/AF)	20 (6.4)	31 (14.2)	0.002
-Alzheimer/Dementia	13 (4.1)	16 (7.3)	0.110
-Osteoporosis	13 (4.1)	7 (3.2)	0.580
Polypharmacy (Use of ≥5 medications)	85 (27.1)	68 (31.2)	0.302
Anti-platelet use	58 (18.5)	64 (29.4)	0.003
Ambulatory status before fracture			<0.001
-Home walk, with gait aids	35 (11.1)	70 (32.1)	
-Home walk, no gait aids	20 (6.4)	40 (18.3)	
-Community walk, with gait aids	16 (5.1)	25 (11.5)	
-Community walk, no gait aids	243 (77.4)	83 (38.1)	
Indoor, falling area	249 (79.3)	184 (84.4)	0.227
Type of fracture			<0.001
-Subtrochanteric	3 (1.0)	5 (2.3)	
-Intertrochanteric	186 (59.2)	75 (34.4)	
-Neck of femur	125 (39.8)	138 (63.3)	
Recurrent fracture	13 (4.1)	15 (6.9)	0.164
Laboratory investigations			
-Hematocrit (%, mean ± SD)	33.8 ± 5.3	31.6 ± 5.4	<0.001
-Glomerular filtration rate (mL/min, mean ± SD)	82.0 ± 49.4	71.9 ± 27.4	0.006
Preoperative medical in hospital factors			
-Acute kidney injury	110 (35.0)	101 (46.3)	0.009
-New diagnosis anemia	96 (30.6)	103 (47.3)	<0.001
-Electrolyte disturbances			
-Hyponatremia	39 (12.4)	37 (17.0)	0.140
-Hypokalemia	46 (14.6)	50 (22.9)	0.015
-Hyperkalemia	5 (1.6)	10 (4.6)	0.040
-Urinary tract infection	52 (16.6)	61 (28.0)	0.002
-Preoperative active heart disease (CHF/AF)	11 (3.5)	12 (5.5)	0.264
-Delirium	11 (3.5)	18 (8.3)	0.018
-Pneumonia	1 (0.3)	12 (5.5)	<0.001
-Coagulopathy	2 (0.6)	3 (1.4)	0.048
-COVID-19 infection	2 (0.6)	6 (2.8)	0.049
**Intraoperative factors**			
Time from admission to surgery (hours, median (IQR))	84 (85)	97.5 (95)	<0.001
Surgery within 72 h from admission	142 (45.2)	63 (2.9)	<0.001
Type of operation			<0.001
-Fixation (multiple screw/plate and screw/intramedullary nail)	149 (47.5)	153 (70.2)	
-Arthroplasty	165 (52.5)	65 (29.8)	
Total surgical time (minutes, mean ± SD)	79.8 ± 37.9	77.3 ± 39.7	0.469
Presence of intraoperative surgical complications	5 (1.6)	13 (6.0)	0.006
Anemia during operation	69 (22.0)	65 (29.8)	0.040
Estimate Blood loss (mL, median (IQR))	150 (200)	150 (100)	0.342
Class III-IV ASA classification	222 (70.7)	199 (91.3)	<0.001
Regional anesthesia	265 (84.4)	166 (76.1)	0.017
Total anesthetic time (minutes, mean ± SD)	114.5 ± 33.8	123.1 ± 34.5	0.004
Presence of intraoperative anesthesia complications	109 (34.7)	103 (47.2)	0.004
**Postoperative factors**			
Postoperative ICU and ventilator use	6 (1.9)	19 (8.7)	<0.001
Postoperative oxygen support	126 (40.1)	120 (55.0)	<0.001
Urinary Cath use in the 2nd day postoperative	154 (49.0)	139 (63.8)	0.001
Presence of postoperative surgical complication	0 (0.0)	23 (10.6)	<0.001
Pain scores on rehabilitation day (scores, median (IQR))	2 (0)	2 (1)	0.064
Anemia due to postoperative blood loss	93 (29.6)	93 (42.7)	0.002
Presence of postoperative medical complications			
-Pressure sore	5 (1.6)	18 (8.3)	<0.001
-Delirium	10 (3.2)	24 (11.0)	<0.001
-Pneumonia	1 (0.3)	8 (3.7)	0.004
-Urinary tract infection	8 (2.5)	22 (10.1)	<0.001
Postoperative electrolyte disturbances			
-Hyponatremia	12 (3.8)	11 (5.0)	0.495
-Hypokalemia	20 (6.4)	27 (12.4)	0.016
-Hyperkalemia	0 (0.0)	5 (2.3)	0.011
Postoperative deep vein thrombosis	2 (0.6)	1 (0.5)	1.000
Postoperative pulmonary embolism	1 (0.3)	0 (0.0)	1.000
Postoperative Atrial fibrillation	0 (0.0)	4 (1.8)	0.028
Functional training postoperatively			
Time to start functional training			<0.001
-Less than 48 h postoperatively	167 (53.2)	84 (38.5)	
-48 h to less than 72 h postoperatively	89 (28.3)	63 (28.9)	
-More than 72 h postoperatively	58 (18.5)	71 (32.6)	
Ambulation training	310 (98.7)	68 (31.2)	<0.001
Total training days (days, median (IQR))	2 (1)	3 (1)	0.001
Length of stay ≥ 14 days	41 (13.1)	66 (30.3)	<0.001

Mann-Whitney U test. Abbreviations AF, atrial fibrillation; ASA, American Society of Anesthesiologists; CHF, congestive heart failure; IHD, ischemic heart disease; VHD, valvular heart disease; IQR, interquartile range; SD, standard deviation.

**Table 3:** Univariable and multivariable risk ratios of preoperative, intraoperative, and postoperative prognostic factors associated with ambulatory and non-ambulatory status at discharge following fragility hip fracture surgery.

	Univariable RR	95% CI	*p*-Value	Multivariable RR	95% CI	*p*-Value
**Preoperative factors**						
Age ≥ 80 years	0.69	0.59–0.80	<0.001	0.97	0.86–1.08	0.558
Comorbidities						
-Hypertension	0.89	0.78–1.04	0.157	1.06	0.96–1.18	0.259
-Chronic kidney disease	0.85	0.73–1.01	0.058	1.01	0.90–1.13	0.892
-Musculoskeletal problems	0.87	0.68–1.11	0.259	0.97	0.82–1.14	0.680
-Cerebrovascular disease	0.55	0.42–0.73	<0.001	0.70	0.53–0.91	0.007
-Anemia	0.84	0.66–1.09	0.193	1.11	0.96–1.28	0.165
-Pulmonary disease	0.83	0.64–1.08	0.168	0.92	0.78–1.08	0.331
-Heart disease (AF/VHD/IHD)	0.64	0.42–0.91	0.013	0.85	0.68–1.06	0.145
-Alzheimer/Dementia	0.75	0.50–1.13	0.167	1.05	0.79–1.39	0.722
Anti-platelet use	0.76	0.62–0.93	0.008	1.08	0.91–1.29	0.356
Ambulatory status before fracture						
-Home walk, with gait aids		reference				
-Home walk, no gait aids	1.00	0.64–1.57	1.000	0.86	0.63–1.18	0.359
-Community walk, with gait aids	1.17	0.73–1.87	0.510	0.78	0.54–1.14	0.208
-Community walk, no gait aids	2.24	1.69–2.95	<0.001	1.12	0.92–1.36	0.266
Recurrent fracture	0.78	0.52–1.16	0.222	1.06	0.87–1.27	0.563
Pre-operative medical in hospital factors						
-Acute kidney injury	0.82	0.70–0.96	0.011	0.96	0.88–1.05	0.422
-New diagnosis anemia	0.74	0.63–0.87	<0.001	0.92	0.84–1.01	0.098
-Electrolyte disturbances						
-Hypokalemia	0.76	0.61–0.95	0.016	0.96	0.81–1.10	0.577
-Hyperkalemia	0.56	0.27–1.14	0.112	0.95	0.62–1.44	0.808
-Urinary tract infection	0.74	0.59–0.91	0.005	0.96	0.84–1.11	0.616
-Delirium	0.63	0.39–1.01	0.054	1.06	0.77–1.45	0.722
-Pneumonia	0.13	0.02–0.84	0.032	0.34	0.06–1.76	0.197
-Coagulopathy	0.84	0.73–0.97	0.015	1.02	0.93–1.11	0.725
-COVID-19 infection	0.42	0.13–1.40	0.157	0.68	0.28–1.67	0.402
-Fever of unknown origin	0.46	0.22–0.98	0.043	0.75	0.47–1.18	0.215
**Intraoperative factors**						
Surgery within 72 h from admission	1.32	1.15–1.51	<0.001	1.06	0.97–1.15	0.168
Arthroplasty operation	1.45	1.26–1.67	<0.001	1.17	1.07–1.29	<0.001
Presence of intraoperative surgical complications	0.46	0.22–0.98	0.043	0.66	0.43–1.02	0.064
Presence of intraoperative anesthesia complications	0.80	0.39–0.94	0.005	0.95	0.87–1.04	0.243
**Postoperative factors**						
Postoperative ICU or ventilator use	0.39	0.20–0.80	0.009	0.96	0.60–1.53	0.865
Postoperative need for oxygen support	0.78	0.67–0.90	0.001	1.08	0.97–1.19	0.149
Urinary Cath use in the 2nd day postoperative	0.79	0.68–0.90	0.001	0.95	0.86–1.04	0.255
Anemia due to postoperative blood loss	0.78	0.66–0.92	0.003	1.05	0.95–1.16	0.349
Presence of postoperative medical complications						
-Pressure sore	0.36	0.16–0.78	0.010	0.95	0.51–1.07	0.870
-Delirium	0.49	0.28–0.81	0.006	0.74	0.51–1.07	0.107
-Pneumonia	0.18	0.03–1.17	0.074	0.41	0.08–2.11	0.287
-Urinary tract infection	0.44	0.24–0.79	0.007	0.80	0.54–1.20	0.287
-Postoperative electrolyte disturbances						
-Hypokalemia	0.70	0.50–0.99	0.041	1.06	0.47–0.91	0.730
Time to start functional training						
-Less than 48 h postoperatively		reference				
-48 h to less than 72 h post operatively	0.88	0.75–1.03	0.117	0.92	0.84–1.02	0.120
-More than 72 h postoperatively	0.67	0.55–0.83	<0.001	0.84	0.73–0.97	0.014
Ambulation training	31.6	12.0–83.2	<0.001	24.10	9.14–63.60	<0.001

Abbreviations AF, atrial fibrillation; CI, confidence interval; ICU, Intensive Care Unit; IHD, ischemic heart disease; mRR, multivariable relative risk; SD, standard deviation; uRR, univariable relative risk; VHD, valvular heart disease.

## Data Availability

The original contributions presented in this study are included in the article. Further inquiries can be directed to the corresponding author.

## Abbreviations

The following abbreviations are used in this manuscript:

AAOS	American Academy of Orthopedic Surgeons
ADLs	activities of daily living
AF	atrial fibrillation
AKI	acute kidney injury
ASA	American Society of Anesthesiologists
BI	Barthel Index
CHF	congestive heart failure
CI	confidence interval
COVID-19	coronavirus disease 2019 (COVID-19) infection
eGFR	estimated glomerular filtration rate
EMR	electronic medical records
FUO	fever of unknown origin
Hct	hematocrit
IHD	ischemic heart disease
IQR	interquartile range
ICU	intensive care unit
ICD	the International Classification of Diseases
LOS	the length of hospital stay
mRR	multivariable risk ratio
NICE	the National Institute for Health and Care Excellence
OR	Odds ratio
QOL	quality of life
SD	standard deviation
uRR	univariable risk ratio
UTIs	urinary tract infections
VHD	valvular heart disease
VIF	variant inflation factor
